# Analysis of Glycan Recognition by Concanavalin A Using Absolute Binding Free Energy Calculations

**DOI:** 10.1021/acs.jcim.4c01088

**Published:** 2024-10-16

**Authors:** Sondos Musleh, Irfan Alibay, Philip C. Biggin, Richard A. Bryce

**Affiliations:** a Division of Pharmacy and Optometry, The University of Manchester, Manchester M13 9PT, U.K.; b Department of Medicinal Chemistry and Pharmacognosy, Faculty of Pharmacy, Jordan University of Science and Technology, P.O. Box 3030, Irbid 22110, Jordan; c Open Free Energy, Open Molecular Software Foundation, Davis, California 95616, United States; d Structural Bioinformatics and Computational Biochemistry, Department of Biochemistry, The University of Oxford, South Parks Road, Oxford OX1 3QU, U.K.

## Abstract

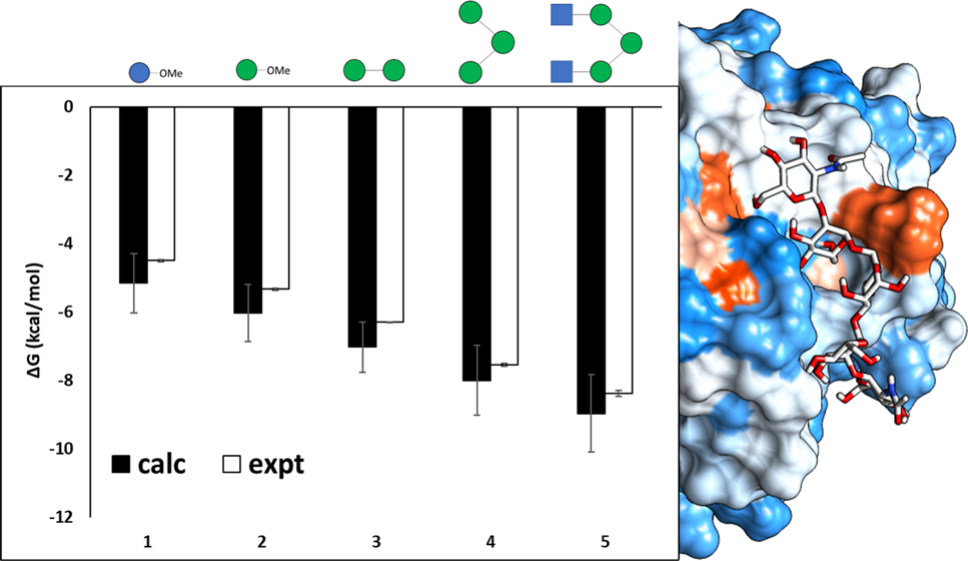

Carbohydrates are key biological mediators of molecular recognition and signaling processes. In this case study, we explore the ability of absolute binding free energy (ABFE) calculations to predict the affinities of a set of five related carbohydrate ligands for the lectin protein, concanavalin A, ranging from 27-atom monosaccharides to a 120-atom complex-type N-linked glycan core pentasaccharide. ABFE calculations quantitatively rank and estimate the affinity of the ligands in relation to microcalorimetry, with a mean signed error in the binding free energy of −0.63 ± 0.04 kcal/mol. Consequently, the diminished binding efficiencies of the larger carbohydrate ligands are closely reproduced: the ligand efficiency values from isothermal titration calorimetry for the glycan core pentasaccharide and its constituent trisaccharide and monosaccharide compounds are respectively −0.14, −0.22, and −0.41 kcal/mol per heavy atom. ABFE calculations predict these ligand efficiencies to be −0.14 ± 0.02, −0.24 ± 0.03, and −0.46 ± 0.06 kcal/mol per heavy atom, respectively. Consequently, the ABFE method correctly identifies the high affinity of the key anchoring mannose residue and the negligible contribution to binding of both β-GlcNAc arms of the pentasaccharide. While challenges remain in sampling the conformation and interactions of these polar, flexible, and weakly bound ligands, we nevertheless find that the ABFE method performs well for this lectin system. The approach shows promise as a quantitative tool for predicting and deconvoluting carbohydrate–protein interactions, with potential application to design of therapeutics, vaccines, and diagnostics.

## 1 Introduction

Carbohydrates serve a number of important biological functions, as energy stores, structural elements, and ligands in a range of recognition processes, including cell–cell and cell–pathogen interactions.^1^ Targeting of carbohydrate-mediated cell–pathogen interactions is a route to the development of small molecule therapeutics, for example, the antiflu neuraminidase inhibitor, zanamivir;^2^ the antidiabetic glucosidase inhibitor, miglitol;^3^ and vaccines, such as those based on the bacterial polysaccharides of*Streptococcus pneumoniae*^4^ and *Haemophilus influenzae*.^5^ Interestingly, glycosylation of proteins can assist pathogens in evading the host immune response but also play a role in stabilizing functional states of the protein, as in the case of the spike protein of SARS-Cov-2, where N-glycans at Asn234 and Asn343 on the spike protein were found to facilitate opening of its receptor binding domain.^6,7^

To guide the design of glycan-related therapeutics, diagnostics, and vaccines, the ability to decipher the structure–activity relationship of a carbohydrate for its receptor protein is key. Computational tools are well placed to analyze carbohydrate–protein interactions in atomistic detail, furnishing energetic components and residue contributions to binding not readily accessible to experiments.^8^ Methods to compute binding free energies from end-point simulations^9−12^ or alchemically^13,14^ have achieved some success in accurate prediction of carbohydrate–protein affinities. For example, a relative binding free energies (RBFE) approach was applied to *R. solanacearum* lectin,^14^ ranking 10 of its monosaccharide ligands with a mean absolute error (MAE) of 1.1 ± 0.1 kcal/mol, including correct prediction of the anomeric preference of d-glucose (Glc) and d-mannose (Man). The RBFE method involves alchemically transforming one ligand into another when protein-bound and unbound; the approach is most suited to studying differences in binding of closely related ligand structures,^15,16^ such as comparing monosaccharide anomers or other epimers, due to the need to keep a conserved common core between end states. Despite recent advances in RBFE methodology,^16,17^ it is still not entirely straightforward to capture the free energy consequences of very large differences in the structure, for example, when comparing carbohydrate ligands that differ in the number of saccharide residues.

However, recent advances in computing absolute binding free energies (ABFEs), by transforming the ligand into a noninteracting species when bound and when in solution, have enabled some success in reliably estimating Δ*G*_bind_ for a range of disparate drug-like ligand structures.^18−20^ A recent meta-analysis studied 853 cases of ABFE calculations of protein–ligand affinities and found that a mean unsigned error in free energy below 3 kcal/mol was achieved in 87% of cases, with an MUE of 1.58 kcal/mol.^21^ Carbohydrate ligands are not particularly drug-like, though, being considerably higher in complexity than small organic molecules: typically, they are larger and more polar, especially in their oligomeric linear or branched forms, with numerous stereogenic centers and rotatable bonds.^22^ Nevertheless, application of ABFEs to computing the protein binding free energies of monosaccharides,^23,24^ disaccharides,^23^ and, in one case, a trisaccharide^25^ has proved encouraging, yielding deviations of 1–3 kcal/mol from the experiment for these systems.

**Figure 1:**
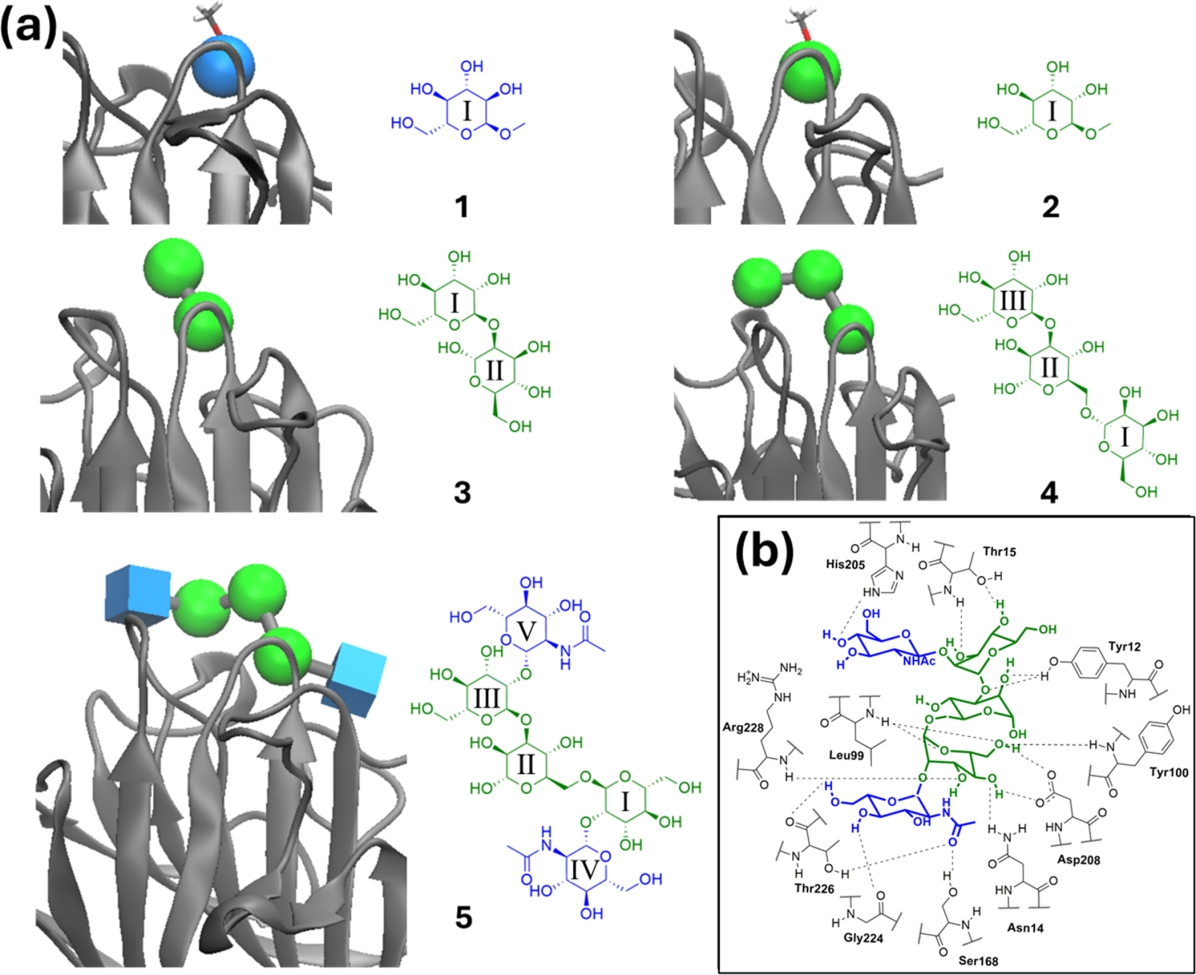
(a) Ligands **1**–**5** and their X-ray bound pose to Con A; these ligands are (**1**) α-MeOGlc, (**2**) α-MeOMan, (**3**) Man-α-(1 → 2)-Man-α-OMe, (**4**) Man-α-(1 → 6)-[Man-α-(1 → 3)]-mannose, and (**5**) β-GlcNAc-(1 → 2)-α-Man-(1 → 3)-[β-GlcNAc-(1 → 2)-α-Man-(1 → 6)]-Man. (b) Polar interactions formed by ligand **5** with Con A in the crystal structure. The high affinity monosaccharide binding site is where ring I binds.

In this case study, we evaluate the ability of ABFE calculations to predict the binding affinities of five carbohydrate substrates to the protein Concanavalin A (Con A). Con A is a glucose/mannose-binding lectin derived from the jack bean (*Canavalia ensiformis*); its carbohydrate complexes have been well-characterized by calorimetry and crystallography and comprise a useful test set for assessing methods for computation of binding affinities.^9−11,13^ Ligands **1**–**5** (Figure 1a) are of increasing complexity, ranging from 27-atom monosaccharides to 120-atom pentasaccharide.

Pentasaccharide **5** has the sequence β-GlcNAc-(1 → 2)-α-Man-(1 → 3)-[β-GlcNAc-(1 → 2)-α-Man-(1 → 6)]-Man and is a common motif of complex-type N-linked glycans. Given its molecular weight of 910 Da, twice that of a large drug-like molecule, and its rather weak binding affinity to Con A of −8.38 kcal/mol,^26^ ligand **5** is a particularly challenging case for ABFE calculations. Here, we assess the performance of ABFE calculations in recovering the structure–activity relationship of pentasaccharide ligand **5** relative to its constituent trisaccharide, Man-α-(1 → 6)-[Man-α-(1 → 3)]-mannose **4**; to a disaccharide, Man-α-(1 → 2)-Man-α-OMe **3**; and to monosaccharide ligand α-MeOMan **2** and its epimer α-MeOGlc **1** (Figure 1a). In their crystal structures with Con A, the residues of ligands **1**–**5** occupy the shallow lectin binding groove to varying degrees (Figure 1a). However, for all five ligands, the high affinity mannose binding site within the groove is occupied, lined by the amino acid residues Asn14, Leu99, Tyr100, Asp208, and Arg228 (ring I for ligands **1**–**5**, Figure 1a,b).

## 2 Materials and Methods

### 2.1 System Preparation and Simulation Details

Initial models for Con A in complex with ligands **1**–**5** were constructed based on the available X-ray structures, with respective PDB entry codes and resolutions of 1GIC (2.00 Å), 5CNA (2.00 Å), 1I3H (1.20 Å), 1CVN (2.30 Å), and 1TEI (2.70 Å).^27−31^ A single subunit of Con A, which can exist as a dimer or tetramer, was retained for the simulations. Protonation and tautomeric states were assigned using MOE 2020.09 consistent with physiological pH.^32^ All crystal waters for this monomer were kept, including the conserved bound water molecule of Con A that is important for the protein’s interaction with ligands **4** and **5**.

Parameters for Con A and its carbohydrate ligands were assigned by using the CHARMM36-feb2021 force field via the CHARMM-GUI tool.^33−37^ Parameters for the Mn^2+^ ion of Con A were modeled based on CHARMM calcium ion parameters, as adopted elsewhere,^38^ given the same charge, coordination pattern, and similar size. All molecular dynamics simulations used the GROMACS 2021.5 software package.^39^ The systems were neutralized with sodium ions and solvated 15 Å beyond the complex using a truncated octahedron with TIP3P water.^40^ The resulting systems contained ∼19,000–22,000 water molecules.

### 2.2 Absolute Free Energy Calculations

The thermodynamic pathway used to calculate the ABFEs follows the protocol of Aldeghi et al.^20,41,42^ Namely, following equilibration, a partial decoupling scheme is employed to follow the alchemical path from a fully interacting protein–carbohydrate complex to a carbohydrate ligand in solution (Figure S1). This partial decoupling scheme involves annihilating ligand partial charges through 11 windows spaced at λ intervals of 0.1 from each other. This is then followed by 21 van der Waals decoupling windows spaced at 0.05 λ intervals. A soft-core potential for decoupled van der Waals interactions was used.^43,44^ Additionally, to restrict ligand motion in the complex, an orientational restraint, as defined by Boresch et al.^45^ was employed and derived using the MDRestraintsGenerator code.^42,46^ This restraint was applied over 12 windows in the complex decoupling phase. In the solvent phase, the influence of this restraint was accounted for analytically;^45^ 31 windows were applied to decouple the ligand from the solvent. Therefore, in total, each ABFE calculation corresponded to 75 window simulations.

Simulations were performed using a stochastic leapfrog integrator^47^ and 2 fs time step. The temperature was controlled by Langevin dynamics,^48^ with a friction constant of 1.00 ps^–1^. LINCS was applied to constrain bonds involving hydrogen, while water molecules were constrained with the SETTLE algorithm.^49^ Periodic boundary conditions were used, with long-range electrostatic and van der Waals interactions treated via the particle mesh Ewald and twin range cutoff schemes, respectively,^50,51^ using a short-range cutoff value of 12 Å and a switching distance of 10.0 Å. Coordinates were stored every 2 ps, while the free energies were calculated every 200 fs.

At each λ window, the systems were independently equilibrated to enable parallelization of the computational workload for a given replica. Thus, for each window, the systems were energy minimized and then heated sequentially from 0 to 298 K over 700 ps under NVT conditions, with a restraint of 1000 kJ/(mol nm^2^) on all atoms of the protein, including metal ions, and the ligand. The systems were then equilibrated under NPT conditions of 1 atm and 298 K in four stages, applying restraints of 1000, 500, and 100 kJ/(mol nm^2^) in the first three stages, respectively, for 200, 200, and 300 ps and without restraints in the final stage of 600 ps. The Berendsen barostat^52^ was applied in the first three stages and the Parrinello–Rahman barostat^53,54^ in the final stage. Following this, a 20 ns NPT production MD simulation was performed at the given λ.

Using the simulation protocol, five replica ABFE calculations were obtained for each protein–ligand complex using independently equilibrated bound poses. For each replica, the initial structure is a frame taken from the preliminary 10 ns MD simulation and represents the structure that is closest to the mean bond, angle, and dihedral values of the restraint used in the ABFE calculations. For the solvent leg, the ligand was extracted from the frame and then solvated, and the solvation free energy calculations were carried out. Estimates of the binding free energies were calculated using the multistate Bennett acceptance ratio (MBAR)^55^ via *alchemical-analysis.py*,^56^ where the first 2 ns from each production window were excluded from the analysis as extra equilibration time. The protein–ligand binding free energy is reported as the average ABFE over these replicas with the associated statistical uncertainty taken as the standard deviation.

In addition to computing absolute free energies of binding and solvation, unbiased MD simulations of each complex were run for 500 ns under NPT conditions for ligands **1**–**5**, following the same set up and equilibration protocol over 2 ns discussed earlier. For hydrogen bond analysis, the *gmx hbond* routine was employed, where heavy atom-heavy atom distance and angle cutoff of 3.5 Å and 30° were used, respectively. Ligand efficiency for computed and experimental binding affinities, LE_calc_ and LE_expt_, respectively, were reported as (*binding free energy*)/(*number of heavy atoms*). Throughout the manuscript, figures were generated using Discovery Studio 2015 (BIOVIA Software Inc.),^57^ VMD, and ChemDraw Ultra 12.0.2.

## 3 Results and Discussion

### 3.1 Estimates of Absolute and Relative Binding Free Energies from ABFE

The absolute binding free energies of carbohydrate ligands **1**–**5** to the lectin protein, Con A, were computed using thermodynamic integration with electrostatic decoupling, based on the available crystal structures of the five complexes. From isothermal titration calorimetry (ITC), the measured binding affinities range from −4.49 kcal/mol for **1** to −8.38 kcal/mol for **5** (Table 1).^26^ The predicted Δ*G*_bind_ values from ABFE calculations spanned a very similar range to experiment, from −5.15 kcal/mol for **1** to −8.97 kcal/mol for **5** (Table 1). There was a modest systematic overestimation of affinity, with a mean signed error over the five ligands of −0.63 kcal/mol (Table 1). Regarding variation across replicas for a given ligand, the highest sampling error was for the largest ligand, **5**, with a standard deviation of 1.13 kcal/mol (Table 1 and Table S1). In accord with the quantitative agreement in binding free energies for **1**–**5**, an accurate estimate of ligand efficiency (LE) is also obtained, with a mean signed error between calculation and experiment of −0.03 kcal/mol per heavy atom (Table 1).

**Table 1:** Standard Binding Free Energies, Δ*G*_bind_ (in kcal/mol) for Carbohydrate Ligands **1**–**5** to Con A from ABFE Calculations (calc) and Isothermal Titration Microcalorimetry (expt)^c^

	**Δ***G*_**bind**_				
**mol**	**calc**	**expt**^**26**^	**Δ*G*_calc–expt_^bind^**	**LE_calc_**	**LE**_**expt**_
**1**	–5.15 (0.87)^a^	–4.49 (0.03)^b^	–0.66	–0.40 (0.07)	–0.35 (0.00)
**2**	–6.03 (0.84)	–5.33 (0.03)	–0.70	–0.46 (0.06)	–0.41 (0.00)
**3**	–7.02 (0.74)	–6.30 (0.02)	–0.72	–0.30 (0.03)	–0.27 (0.00)
**4**	–8.00 (1.02)	–7.54 (0.05)	–0.46	–0.24 (0.03)	–0.22 (0.00)
**5**	–8.97 (1.13)	–8.38 (0.08)	–0.59	–0.14 (0.02)	–0.14 (0.00)
***MSE***			***–0.63***	***–0.03***	

a Calculated errors are standard deviations for five replicate ABFE calculations.

b Experimental errors are those reported in ref (26), namely, the standard deviation of fit between the binding curve from the isothermal titration calorimetry and the calculated curve obtained with the fitted thermodynamic parameters.

c Calculated values represent mean of five replicate ABFE calculations, each using 75 λ windows of 20 ns width. Ligand efficiency for computed and experimental binding affinities, LE_calc_ and LE_expt_, respectively, was reported as kcal/mol per heavy atom. Errors are stated in parentheses, and mean signed error (MSE) is given for binding free energies across ligands and for LE_calc-expt_.

**Figure 2:**
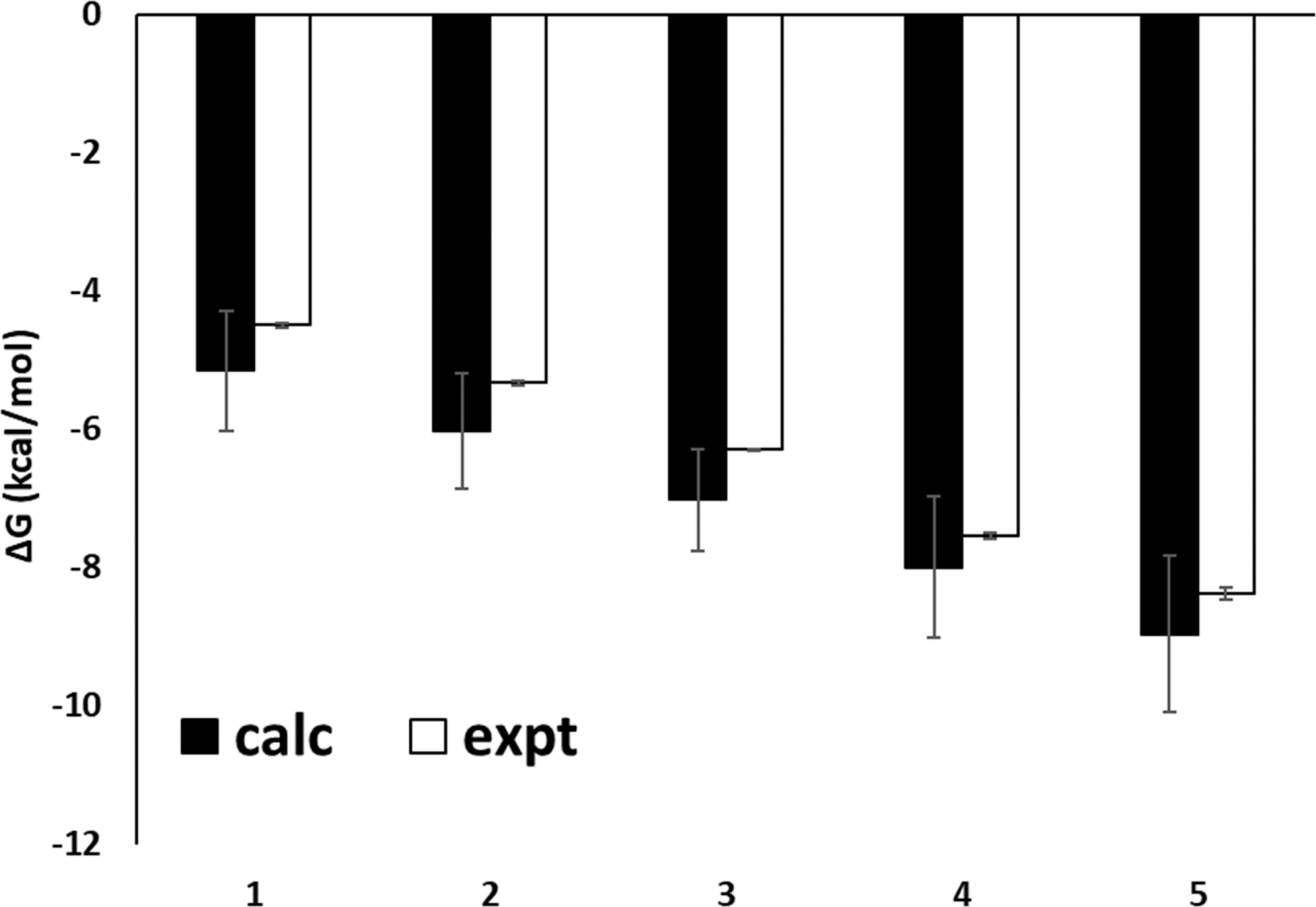
Absolute binding free energies for carbohydrate ligands **1**–**5** to Con A from ABFE calculations (black) and the experiment (white). Energies are given in kcal/mol. Correlation coefficient, MSE, and RMSE were calculated from mean estimate values, with error bars obtained as the standard deviation of the means generated through bootstrap resampling (1000 iterations).

**Figure 3:**
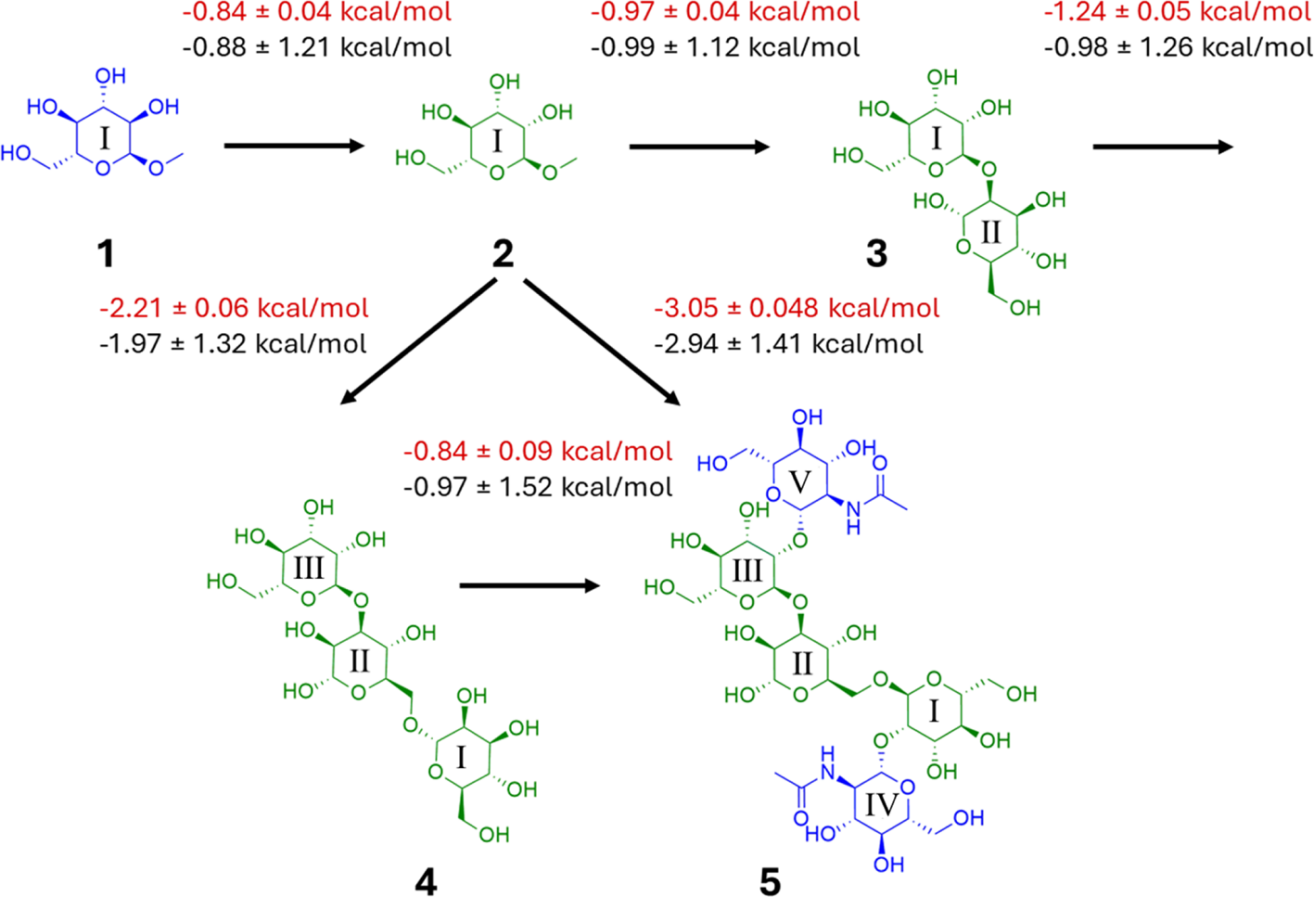
Selected comparative binding free energies of Con A with saccharide ligands **1**–**5**, and corresponding standard deviations across replicates, from the experiment (red)^**26**^ and estimated from ABFE calculations (black).

Given the close agreement in the computed and observed absolute values of binding free energy, a strong correlation between the calculated and experimental binding affinities of the complexes was also found (Figure 2). The associated Pearson coefficient *r* and Kendall tau coefficient are effectively unity, albeit for a data set of only five ligands. The relationship between binding free energy and saccharide structure appears to be reproduced well: for the stepwise progression from ligands **1**–**5**, corresponding in three of the steps to significant differences in the ligand structure and size, the MAE in computed ΔΔ*G*_bind_ is 0.11 kcal/mol with respect to the experiment (Figure 3). The internal variation in ΔΔ*G*_bind_ estimates (i.e., combined errors for replica-based estimates reported in Figure 3) is larger in magnitude than this MAE, ranging from 1.12 kcal/mol for **2**→ **3** to 1.52 kcal for **4**→ **5**. The predicted ΔΔ*G*_bind_ values from ABFEs considerably improve upon estimates of ΔΔ*G*_bind_ furnished by a recent MM/PBSA-based study of Con A complexes,^11^ which obtained an MAE in ΔΔ*G*_bind_ for the same ligand comparisons of 7.28 kcal/mol. Although here a smaller set of ligands are considered, this error in ΔΔ*G* compares well with an MAE of ∼1 kcal/mol from lectin RBFE estimates of 10 monosaccharides.^14^ The associated sampling error in the latter study, however, is smaller, given the RBFE protocol and more modest changes in ligand structure studied, with a value of 0.06 kcal/mol.

### 3.2 Structure–Activity Relationships

**Figure 4:**
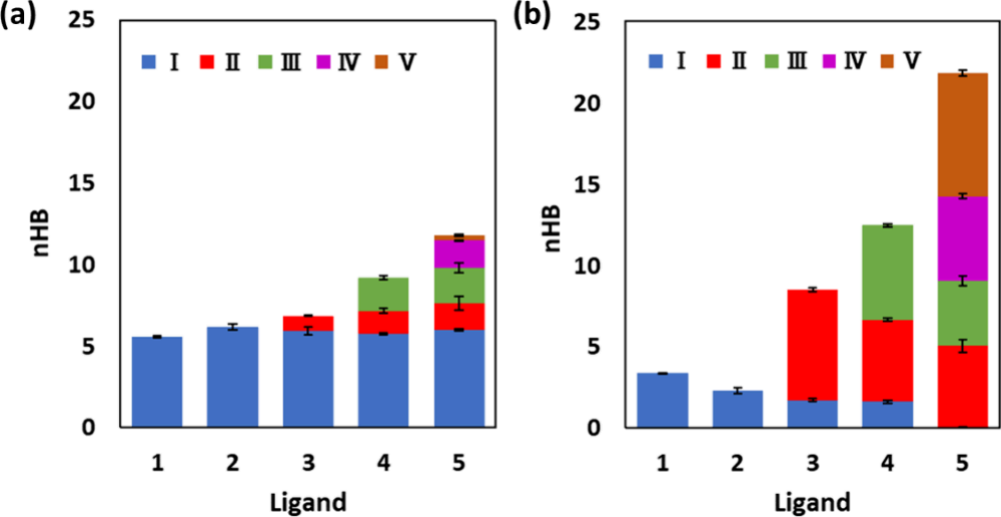
Number of (a) protein–ligand and (b) solvent–ligand hydrogen bonds, *n*_HB_, for rings *I*–*V* of ligands **1**–**5**, averaged over 500 ns molecular dynamics simulation. Error bars are derived from block averaging.

We now consider in more detail the ability of ABFE calculations to capture substrate differences in carbohydrate binding to Con A. First, we compare the closely related monosaccharides α-MeOGlc **1** and α-MeOMan **2** (Figure 3), which differ only by a change in epimeric configuration at the C2 position. Both ligands bind in the same pose to the high affinity monosaccharide binding site of Con A, formed by residues Asn14, Leu99, Tyr100, Asp208, and Arg228 (Figure 1 and Figure S2). The key difference in binding mode is that, for **1**, the equatorial 2-OH projects out into solution, whereas in **2**, the axial 2-OH interacts with the protein. Correspondingly, **2** is favored by 0.84 ± 0.04 kcal/mol over **1** experimentally (Table 1). ABFE calculations provide a ΔΔ*G*_bind_ estimate of 0.88 ± 1.12 kcal/mol (Figure 3); this mean value indicates the correct preference, although with a significant standard deviation. For further insight, we also performed a 500 ns unbiased MD simulation of the complexes of Con A with **1** or **2** bound (Supporting Information, Figures S3 and S4). As expected, these simulations indicate greater overall hydrogen bonding to Con A of **2** over **1** (Figure 4); this mainly arises from 0.46 more hydrogen bonds on average made by the axial 2-OH group of **2** (Table S2).

With a calorimetric Δ*G*_bind_ of −5.33 kcal/mol, monosaccharide α-MeOMan **2** is the key anchoring residue within larger oligosaccharide forms of complex-type N-linked glycans such as ligand **5**. Consequently, the addition of a second mannose residue at the reducing position of **2**, to give α-(1 → 2)-linked dimannoside **3**, results in only a modest benefit in Δ*G*_bind_ by 0.97 ± 0.04 kcal/mol experimentally (Figure 3). Again, ABFE calculations correctly indicate only a small improvement in binding due to this change with an estimate of 0.99 ± 1.12 kcal/mol. Unbiased MD simulation of the complex indicates hydrogen bonding made by the additional ring of **3** (Figure 4 and Figure S5).

The core trimannose ligand, Man-α-(1 → 6)-[Man-α-(1 → 3)]-mannose **4**, is experimentally observed to bind with 1.24 ± 0.05 kcal/mol higher affinity than disaccharide **3**. ABFE calculations predicted this change to be 0.98 ± 1.26 kcal/mol. Ligand **4** makes favorable hydrogen bonds with Pro13 and Thr15 of Con A (Figure S6); the improved interactions appear facilitated by the change in the glycosidic linkage from α-(1 → 2) for **3** to α-(1 → 6) for **4** (Figure 1 and Figure S2).

Addition of terminal β-(1 → 2)-GlcNAc arms to the core mannoside **4** yields pentasaccharide **5**. The unusually small decrease in observed Δ*G*_bind_ by 0.84 ± 0.09 kcal/mol accompanying this modification is captured well by the ABFE method, yielding a computed value of 0.97 ± 1.52 kcal/mol (Figure 3). The corresponding reduction in ligand efficiency on proceeding from **4** to **5** is predicted as 0.10 ± 0.04 kcal/mol per heavy atom, compared with an experimental value of 0.08 ± 0.00 (Table 1). This drop in LE is observed despite the additional protein contacts formed by one of the β-GlcNAc residues of **5** (ring IV in Figure 3), with amino acid residues Ser168, His205, Gly224, Thr226, and Arg228 (Figure 1b). The second β-GlcNAc unit of **5** (ring V, Figure S7) projects out into solution and contributes negligibly to protein–ligand hydrogen bonding (Figure 4). It has been pointed out, however, that the polar interactions of ring IV with Con A are not optimal.^9^ ABFE calculations appear able to correctly capture the minimal contribution of ring IV to the binding affinity of pentasaccharide **5**. Weak hydrogen bonding to Con A by ring IV of **5** is also evidenced by the low population and frequent transitions of hydrogen bond interactions involving this ring over the 500 ns unbiased MD simulation of the **5**/Con A complex (Figure S7).

While the progression from **1** to **5** described above involves only the modest changes in experimental binding free energy, larger changes in Δ*G*_bind_ are also captured well by the ABFE calculations. For example, the addition of two or four residues to monosaccharide **2**, i.e., **2** → **4** and **2** → **5**, results in a reduction in Δ*G*_bind_ measured by ITC, of 2.21 ± 0.06 and 3.05 ± 0.08 kcal/mol, respectively (Figure 3 and Table 1). The corresponding ABFE values of 1.97 ± 1.32 and 2.94 ± 1.41 kcal/mol reproduce well the direction and magnitude of these free energy changes. These computed changes for **2** → **4** and **2** → **5** yield LE reductions of 0.22 ± 0.07 and 0.32 ± 0.07 kcal/mol per heavy atom, matching well the corresponding experimental values of 0.19 ± 0.00 and 0.27 ± 0.00 kcal/mol per heavy atom. This level of fidelity in relative free energies and LE from ABFE calculations is comparable with an MAE of ∼1 kcal/mol in ΔΔ*G*_bind_ from RBFE estimates of monosaccharide-lectin affinities.^14^ In a study of Con A lectin specifically,^11^ MM/PBSA-based affinities for ligands including **1**–**5** did not obtain quantitative agreement with the experiment: particularly notable is the prediction of **4** → **5** as −12.10 kcal/mol (with a standard error of 4.95 kcal/mol),^11^ as opposed to only −0.84 ± 0.09 and −0.97 ± 1.52 kcal/mol from ITC and ABFEs, respectively (Figure 3).

### 3.3 Analysis of Errors in ABFE Calculations

**Figure 5:**
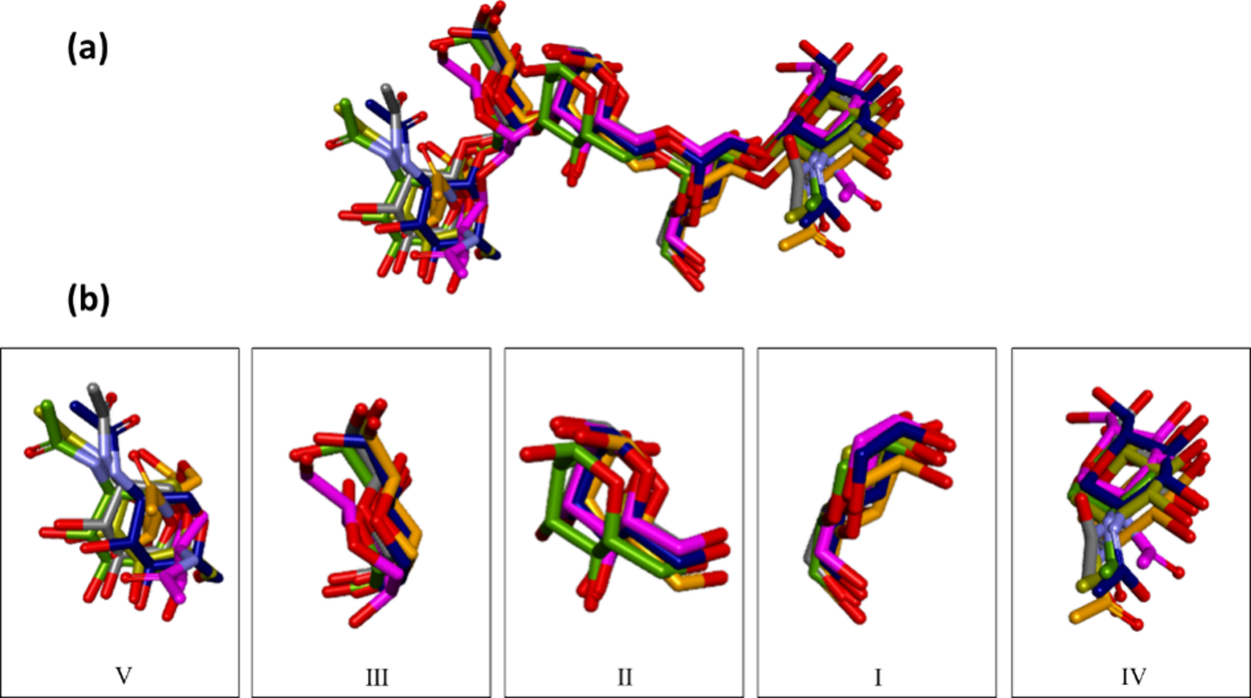
(a) Starting frames of five replicas of ligand **5**, superposed onto the crystal pose based on protein all atoms RMSD. (b) Detail of rings *I*–*V* of ligand **5** from this superposition of replicas. Carbon atoms of ligand **5** in replicas *1*–*5* are colored magenta, orange, gold, navy blue, and gray, respectively, while those of the crystal structure are colored green. Hydrogen atoms removed for clarity.

As discussed above, it appears that ABFE estimates can discriminate the key anchoring residue of pentasaccharide **5** (ring I) from residues that contribute modestly to binding (rings II and III) and those that contribute negligibly to affinity (rings IV and V). While this structure–activity relationship is encouraging, we turn now to comment on potential sources of errors associated with these ABFE estimates, given that ligands **1**–**5** are large, flexible, weakly binding ligands. As noted above, the sampling error, estimated through calculating the standard deviation over the five replica ABFE calculations for ligands **1**–**5**, ranges from 0.74 to 1.13 kcal/mol, with an average of 0.92 kcal/mol (Table 1). These values are considerably higher than the standard deviations associated with the free energies from ITC measurements, which range from 0.02 to 0.08 kcal/mol; these values are very low indeed, when considered in the context of an experimental reproducibility survey, which indicated an experimental root-mean-square error in free energies on average of ∼1 kcal/mol.^58^ Perhaps unsurprisingly, the highest uncertainty in prediction is for the largest ligand, pentasaccharide **5**, with a standard deviation and range across replicas of 1.13 and 2.76 kcal/mol, respectively (Table 1 and Table S1). Although initiated from the same crystallographic structure, the five replicas of Con A/**5** complex equilibrate over the 10 ns preliminary MD simulation to slightly different conformers for each replica (Figure 5a).

**Figure 6:**
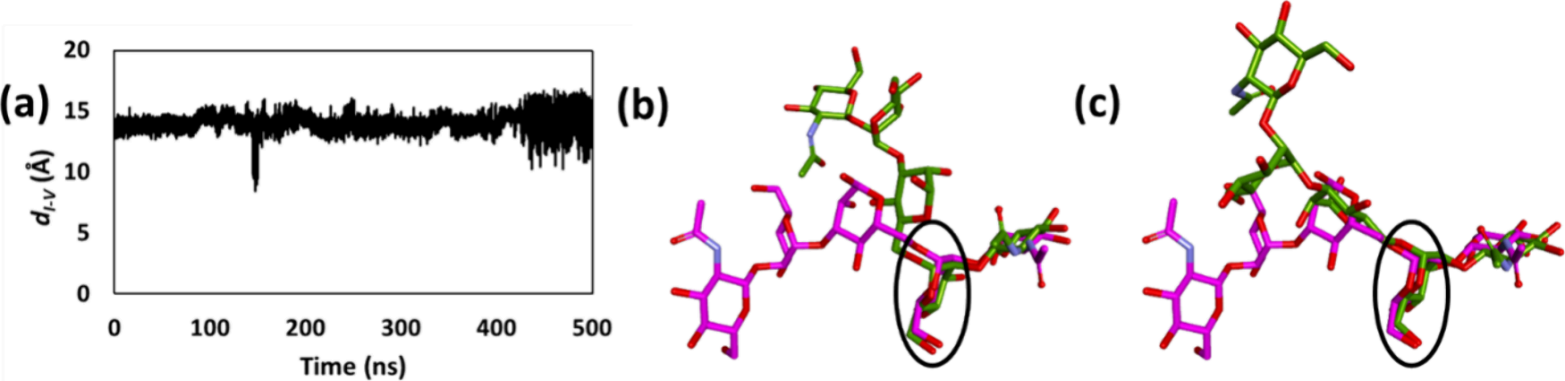
(a) Time series of distance *d*_*I–V*_ between O5 atoms of terminal GlcNAc residues of ligand **5**. Snapshots (in green) of compound **5** MD simulation at (b) 149 and (c) 490 ns, superposed with crystallographic pose of ligand **5** (in magenta). The well-preserved key anchoring mannose residues are labeled by black circles.

To some degree, as might be expected both computationally and experimentally, the lower the LE of the saccharide residue, the larger the structural variation observed in its bound pose: the closest similarity in conformation is found for the anchoring mannose residue, ring I, in the monosaccharide binding site (Figure 5b). Furthermore, the unbiased 500 ns simulation of the Con A/**5** complex indicated periodic significant changes in ligand pose, as indicated by distance *d*_*I–V*_ between the O5 ring atoms of the terminal GlcNAc residues (Figure 6a); and by snapshots taken at 149 and 490 ns superposed onto the crystal structure (Figure 6b,c). Interestingly, the key anchoring mannose (ring I) remained firmly attached to Con A throughout the trajectory, as anticipated from its network of favorable interactions with the protein.

**Figure 7:**
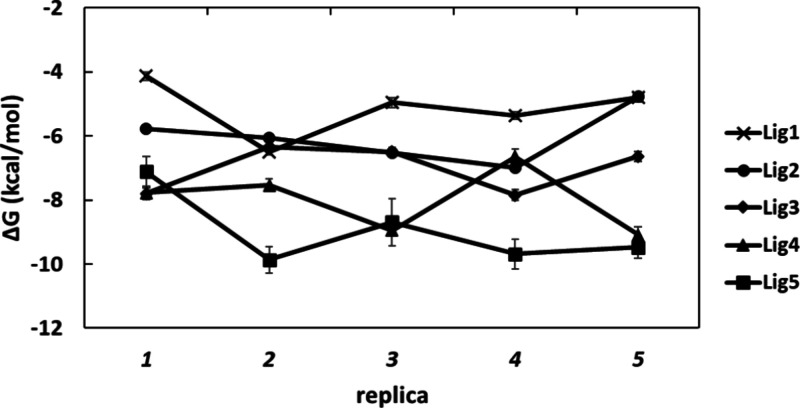
Variation in binding free energies calculated for the five replicas of each of the complexes (in kcal/mol). Error bars are from MBAR estimate.

**Figure 8:**
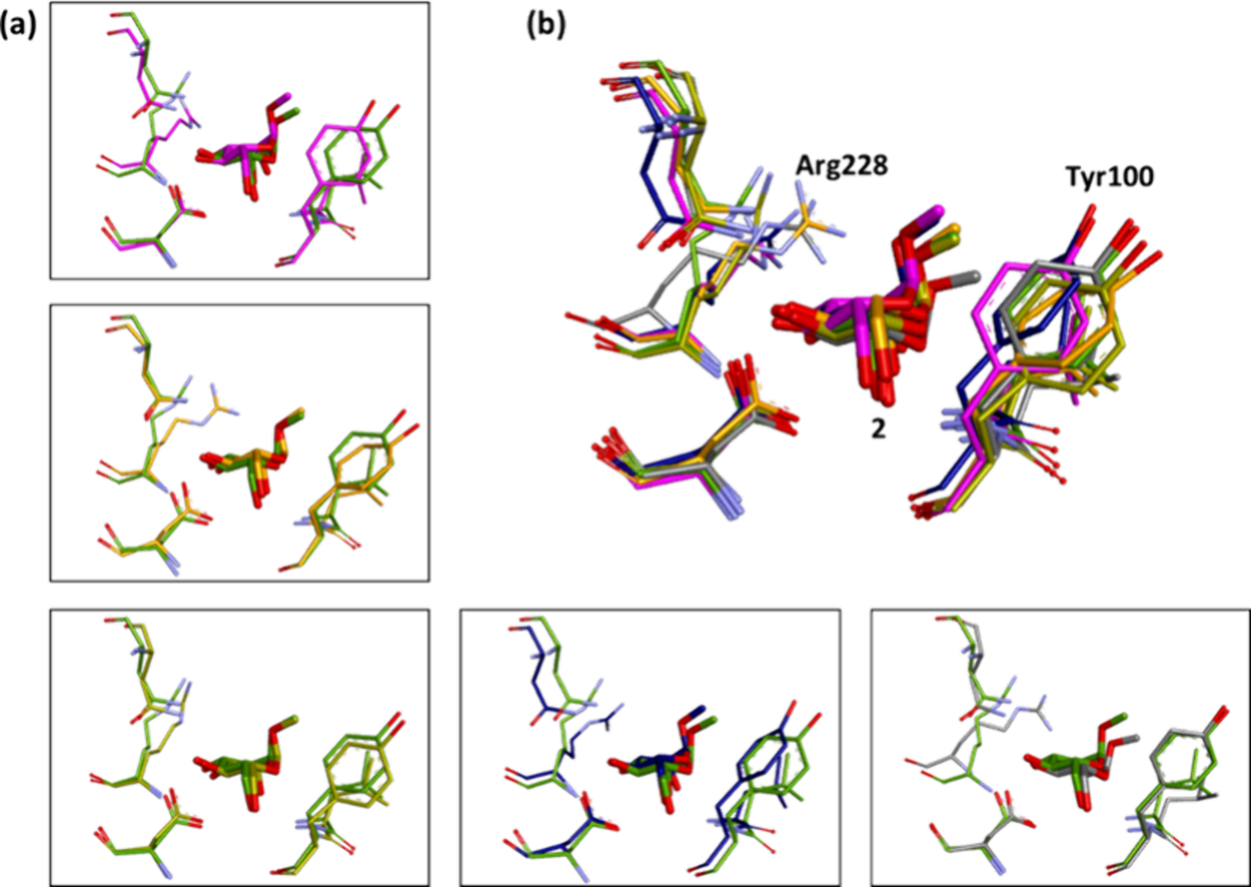
(a) Starting structures of replicas *1*–*5* for ligand **2**, each separately superposed on to the crystal structure. (b) Starting structures of replicas *1*–*5* superposed together on to the crystal structure. Carbon atoms of crystal pose are colored green, while replicas *1–5* are magenta, orange, gold, navy blue, and gray, respectively. Hydrogen atoms are omitted for clarity.

While the range in Δ*G*_bind_ across replicas is highest for pentasaccharide **5**, with a value of 2.76 kcal/mol, a somewhat lower but non-negligible range of 1.51–2.44 kcal/mol is found for ligands **1**–**4** (Figure 7 and Table S1). Even for anchoring monosaccharide **2**, with the highest LE of the ligands (Table 1), computed Δ*G*_bind_ values over the five replicas range from −4.77 for replica *5* to −6.99 kcal/mol for replica *4* (Figure 7 and Table S1); this indicates incomplete convergence, which is expected given the flexible nature of these ligands. The variation over replicas is associated with the protein–ligand rather than unbound ligand legs of the free energy cycle (Figure S8). We observe that the overall bound pose of **2** is well preserved across the starting structures of replicas *1–5* (Figure 8); however, some hetereogeneity in Con A amino acid residue orientation and proximity is evident, mainly for Tyr100 and Arg228 (Figure 8). A similar variation in the local protein environment is found for the other four ligands in their equilibrated structures (Figure S9).

Finally, we also evaluate the effect of the initial crystal structure on the calculated binding free energies. Different initial crystallographic structures of the Con A monomer complex with substrates **2** and **5** were examined. In the preceding calculations, chains D and C were used as the initial structures to compute the ABFE for ligands **2** and **5**, respectively, as those chains displayed the fewest geometric outliers in key binding site residues.^28,31^ For comparison, we used chains A and B to calculate the binding free energies of ligands **2** and **5**, respectively, employing five replicas per complex (Table S3). This yielded a calculated ABFE of −7.07 ± 0.73 kcal/mol for ligand **2**, with a signed error of −1.74 kcal/mol relative to the experiment. For ligand **5**, the computed Δ*G*_bind_ was −10.70 ± 1.37 kcal/mol, with a signed error of −2.32 kcal/mol.

**Figure 9:**
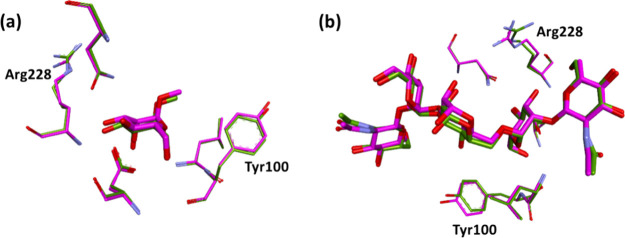
(a) Crystal structures of chain A (green) and D (magenta) of ligand **2**/Con A complex superposed; side-chain torsion angles differ by up to 6° for Arg228 and 20° for Tyr100. (b) Crystal structures of chain B (green) and C (magenta) of the ligand **5**/Con A complex superposed; side-chain torsion angles differ by up to 27° for Arg228 and 7° for Tyr100.

These deviations are somewhat higher than those found for ABFE estimates based on the higher quality chain D and C structures, where the mean signed errors for ligands **2** and **5**, respectively, were −0.72 and −0.59 kcal/mol (Table 1). Comparing the X-ray structures of the complexes, the poses of the ligands are well preserved in the corresponding pairs of chains, but subtle differences were observed for the crystallographic coordinates of the interacting amino acids, particularly, Arg 228 and Tyr100 (Figure 9). Indeed, the Arg228 side chain did not appear to fit well to the electron density in chains A and B of ligands **2** and **5** complexes, respectively. We note that the uncertainty in the calculated ABFEs, estimated as the standard deviation over the five replica ABFE calculations, gets smaller as the resolution of the crystallographic structure improves (Table S4); however, the increased complexity of sampling the interactions of larger ligands undoubtedly also plays a role in determining the ABFE error.

## 4 Conclusions

Here, we have demonstrated the ability of an ABFE approach to quantitatively predict the absolute binding free energies of monosaccharide, disaccharide, trisaccharide, and pentasaccharide ligands to the lectin, Con A. The errors in the relative affinity of these ligands are 0.11 kcal/mol from ABFE calculations, which are considerably improved on those obtained from MM/PBSA-based calculations for these complexes (7.28 kcal/mol) and are comparable in accuracy with free energy estimates for much smaller changes in the (mono)saccharide structure using a RBFE method.^14^ However, for single or hybrid topology RBFE approaches, which typically rely upon close analogy in binding pose and the avoidance of ring breaking or formation,^16^ computing a two-residue alchemical deletion on mutation from ligand **5** to **4** would be challenging to converge.

The error in an absolute free energy of 0.63 kcal/mol found here is smaller than that observed in preceding ABFE studies for smaller carbohydrate ligands, where deviations from experiment ranged from 1 to 3 kcal/mol.^23−25^ In this comparison, we note the effect of force field choice on estimated Δ*G*_bind_: for a study of ABFEs for up to seven monosaccharide/disaccharide–protein systems by Plazinska and Plazinski,^23^ use of the CHARMM36 force field for the ligand and protein^59^ provided an improved correlation with experimental binding free energies over an AMBER GLYCAM06-j/ff99SB-ILDN potential,^60^ although deviations in absolute error in these two calculations were on the order of 3 kcal/mol. In both cases, the TIP3P water model was used, as is the case in the current study. In this work, we use the CHARMM26-feb2021 force field for the carbohydrate and protein, obtaining an error of 0.63 kcal/mol, which lies at the lower end of the expected range in error for ligand–protein ABFEs: a recent meta-analysis of 853 protein–ligand absolute binding free energy calculations from 34 different research groups found a MUE of 1.58_1.34_^1.83^ kcal/mol with a variance of 1.71_1.37_^2.06^ kcal^2^/mol^2^ (here indicating the 95% confidence intervals).^21^ These calculations, which include both alchemical and geometrical approaches (e.g., attach-pull-release and confine-and-release^61^), indicate both accuracy and precision in drug-like ligand–protein ABFEs.

As large, flexible ligands with low ligand efficiency, adequately sampling the bound poses of carbohydrate ligands is indeed demanding. Ligand flexibility and large binding site conformational space have been noted as challenges for ABFE protocols previously,^62^ for example, in computing absolute binding affinities for ligands of the MCL-1 receptor.^42^ Conformational sampling via several independent replicas, of tens of nanoseconds per window, to obtain more reliable free energy estimates appears to be of particular importance for carbohydrates, which typically bind to their protein receptors with modest affinities. For the pentasaccharide ligand **5** in particular, we observe long time scale conformational events over a 500 ns MD trajectory; adequately sampling those states within the numerous λ windows of ABFE calculations is a formidable prospect. We also find that subtle differences in ligand–protein pose appear to contribute to some variation in the calculated free energy of binding. This observation was true both for intrareplica variation in Δ*G*_bind_ estimates for a given chain, and for differences in replica predictions for different choices of chain coordinates.

Nonetheless, the ability of the ABFE method to furnish predictive estimates of ΔΔ*G*_bind_ for carbohydrate ligands such as **5** and its constituent residues, represented by ligands **2** and **4**, is valuable, offering a powerful tool for quantitatively dissecting the residue-wise affinity of these oligosaccharide–protein complexes and identification of binding hot spots. These significant changes in LE are closely reproduced: the experimental LEs for monosaccharide **2** (−0.41 kcal/mol per heavy atom), trisaccharide **4** (−0.22), and pentasaccharide **5** (−0.14) are predicted as −0.46 ± 0.06, −0.24 ± 0.03, and −0.14 ± 0.02 kcal/mol per heavy atom, respectively. Thus, the ABFE approach successfully discerned the anchoring role of the key mannose residue (ring I) from two moderately bound mannose residues (rings II and III); and from a bound β-GlcNAc residue (ring IV) that makes crystallographic contacts with Con A but provides almost no benefit in binding free energy.

In terms of the computational expense of conducting these calculations, we performed protein–ligand decoupling simulations on four Nvidia A100 GPUs for the pentasaccharide **5**/conA complex; and on six Nvidia V100 GPUs for the remaining four ligand/ConA complexes. Typically, one complete ABFE calculation for **5**/ConA would require 4 days of wall-clock time, and ConA complexes of **1**–**4** would use 2–3 days of wall-clock time. Alongside protein–ligand calculations on GPUs, ligand–water decoupling legs were run using 256 Intel Skylake cores and were completed within a day. The overall timings indicate that five replica ABFE calculations for a ligand/protein complex represent a significant but not impractical investment of computational time. This cost could be further reduced by optimization of the λ schedule, for example, through the scheme of thermodynamic trailblazing.^63^

We also note here that while Con A provides an informative, well-characterized model for a range of carbohydrate–protein interactions, this case study represents only a subset of the types of glycan–protein contacts possible. For example, lectins such as the mannose binding protein and LecB from *P. aeruginosa* feature direct Ca^2+^–carbohydrate interactions in the binding site. This contrasts with Con A, where the two divalent metal cations present in the protein provide a scaffolding function for the protein’s architecture but do not interact directly with ligands. In a similar way, multiple CH−π interactions are frequently a feature of carbohydrate–protein recognition^64,65^ However, stacked interactions of the aromatic residues of ConA and its saccharide ligands are not observed from crystallography or simulation.^66^ Interestingly, in the aforementioned ABFE work by Plazinska and Plazinski,^23^ the seven monosaccharide/disaccharide–protein complexes studied feature various aromatic residue–carbohydrate stacking arrangements. While a reasonable correlation between experimental and computed binding free energies was observed, quantitative agreement in absolute values was not found, with the authors concluding that further force field optimization may be required. For calcium-dependent lectins, capturing saccharide binding affinities involving direct metal–carbohydrate interactions is an even greater challenge: using a fixed charge force field to reproduce observed crystal structures of LecB complexes, for example, which feature two closely positioned calcium ions directly interacting with Lewis X tetrasaccharide, required judicious reparameterization of the metal ions and binding site carboxylate groups.^67^ Nevertheless, ABFE calculations in the current work have proved quantitative in resolving residue contributions for the case of oligosaccharide binding to Con A. With advances in potential energy functions and sampling methods, absolute binding free energy protocols have the potential to decipher the molecular recognition of a wide variety of glycan–protein systems, as a prelude to the design of potential new therapeutics, diagnostics, and vaccines.

## Data Availability

Input topologies, coordinates, and simulation control files are provided at https://zenodo.org/records/12530173. The implementation of the MDRestraintsGenerator can be found at https://zenodo.org/records/6977294. Estimates of the binding free energies were calculated using *alchemical-analysis.py* (https://github.com/MobleyLab/alchemical-analysis). Structural and energetic analyses of carbohydrate-Con A interactions from MD simulations and ABFE calculations are available in Supporting Information. Figures were generated using Discovery Studio 2015 (https://www.3ds.com/products/biovia) and ChemDraw Ultra 12.0.2 (https://revvitysignals.com/products/research/chemdraw).

## Supporting Information Available

Structural and energetic analyses of carbohydrate-Con A interactions from MD simulations and ABFE calculations (PDF)
SMILES strings for ligand structures (PDF)
