# Supplementary material for: Analysis of Glycan Recognition by Concanavalin A Using Absolute Binding Free Energy Calculations
Source: J Chem Inf Model. 2024 Oct 16;64(20):8063–73. doi: 10.1021/acs.jcim.4c01088 (PMC11523069; doi:10.1021/acs.jcim.4c01088)
Supplement: Supplementary file 1 — ci4c01088_si_001.pdf [file ci4c01088_si_001.pdf]

Supporting Information

Analysis of glycan recognition by concanavalin A using absolute binding free energy calculations

Sondos Musleh,[a,b] Irfan Alibay,[c,d] Philip C. Biggin,[d] and Richard A. Bryce[a]

[a] *Division of Pharmacy and Optometry, The University of Manchester, Manchester, M13 9PT, UK.*

[b] *Department of Medicinal Chemistry and Pharmacognosy, Faculty of Pharmacy, Jordan University of Science and Technology, P.O. Box 3030, Irbid, 22110, Jordan*

[c] *Open Free Energy, Open Molecular Software Foundation, Davis, California 95616, United States*

[d] *Structural Bioinformatics and Computational Biochemistry, Department of Biochemistry, The University of Oxford, South Parks Road, Oxford, OX1 3QU, UK.*

Figure S1 Thermodynamic cycle used to calculate binding free energies throughout this work represented by conA/α-MeOMan complex (PDB code 5CNA). The left column represents simulations involving ligand in solution, while right column represents simulations of protein-ligand system. The red circle and the paper clip represent restraints, and grey coloured ligand indicates a non-interacting ligand. Thermodynamic states considered are: **(a)** ligand and protein free in solution, **(b)** ligand annihilated from solution, **(c)** annihilated restrained ligand, **(d)** annihilated restrained ligand placed in protein cavity, **(e)** restrained ligand interacting with protein, and **(f)** bound unrestrained ligand.

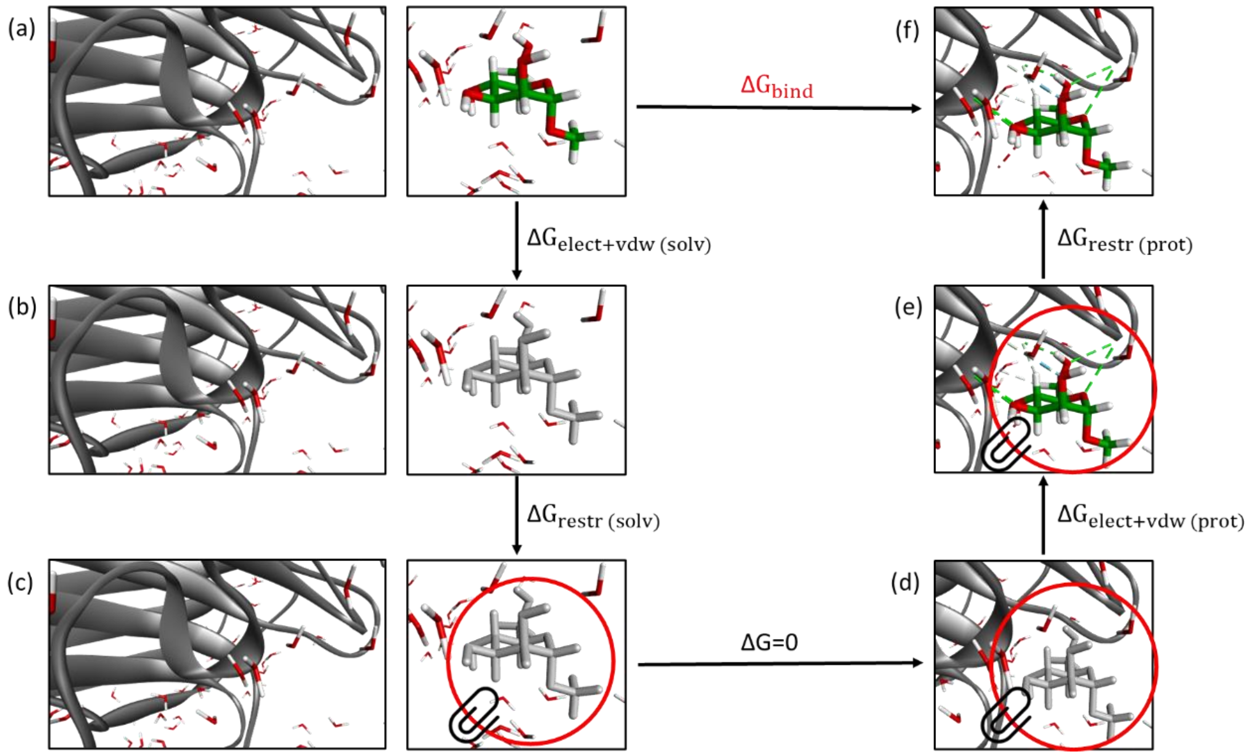

Figure S2 Polar interactions of ligands **1** – **5** with conA featuring in their crystal structures.

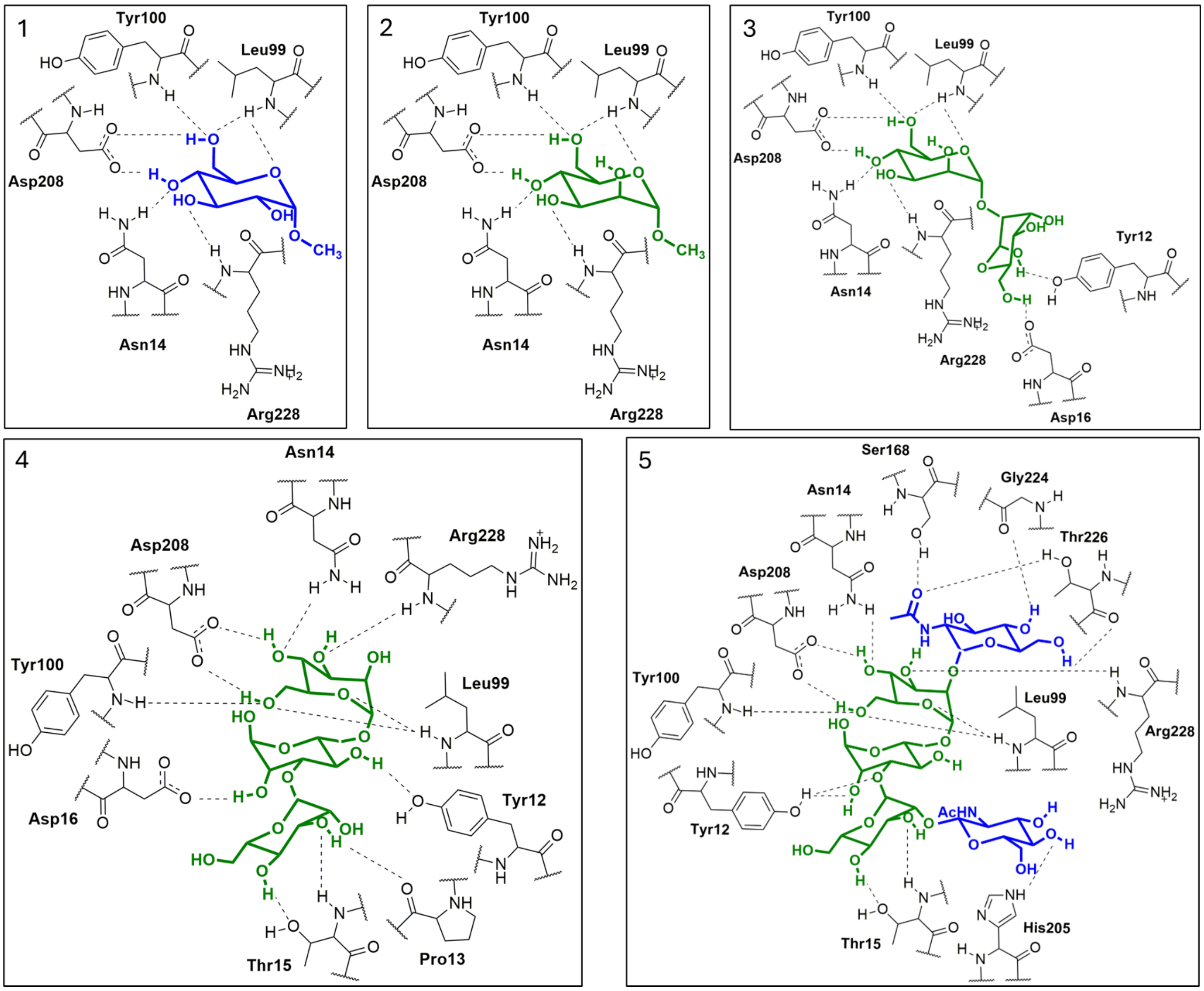

Figure S3 Time series for distance between selected atoms of α-MeOGlc (ligand **1**) and conA over 500 ns MD simulation.

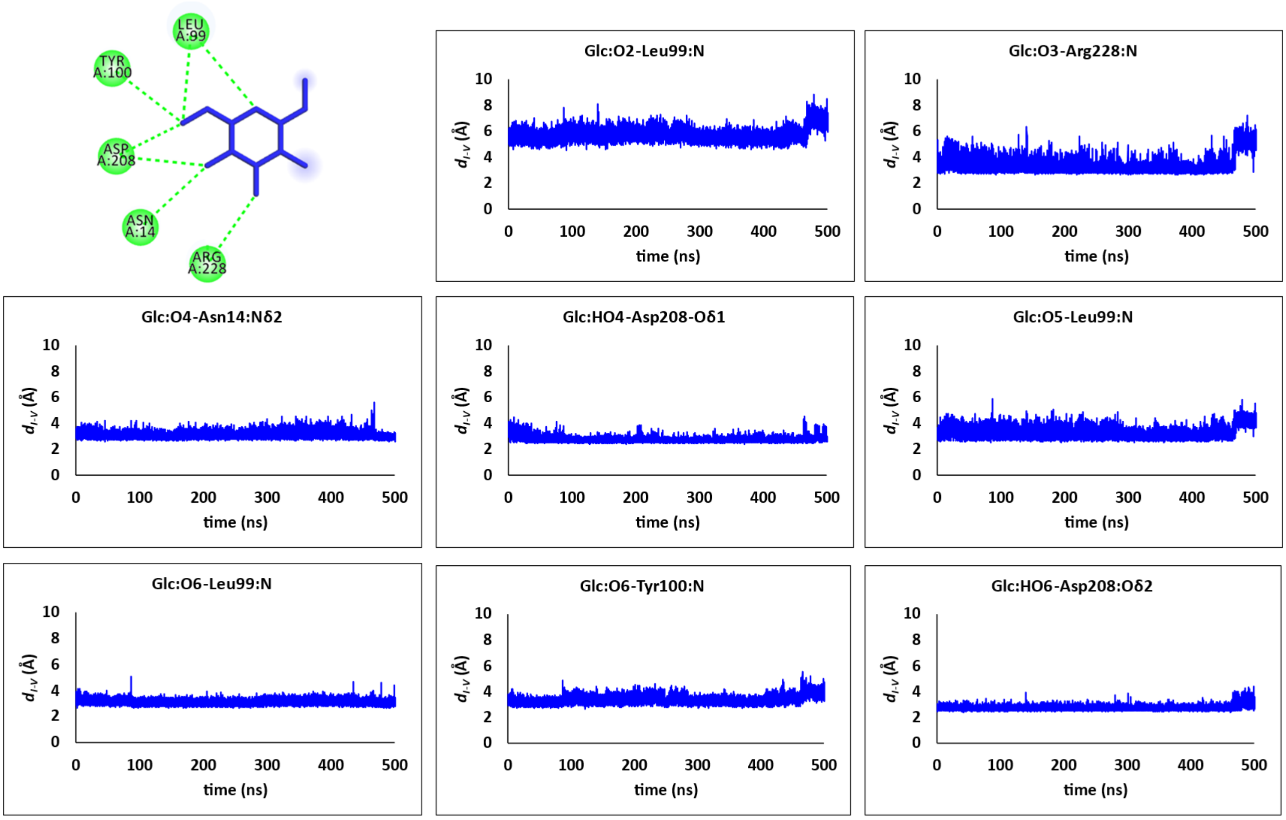

Figure S4 Time series for distance between selected atoms of α-MeOMan (ligand **2**) and conA over 500 ns MD simulation.

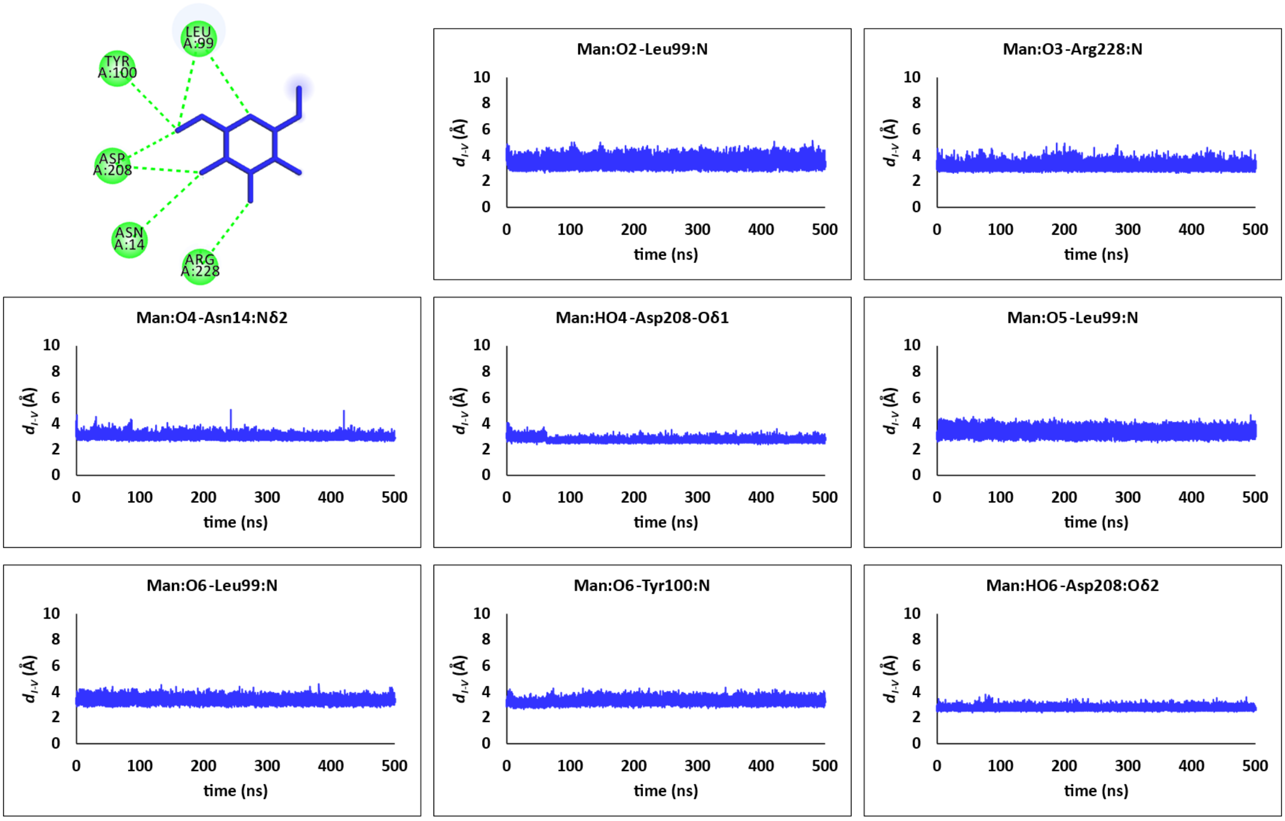

Figure S5 Time series for distance between selected atoms of -α-(1→2)-Man-α-OMe (Ligand **3**) and conA over 500 ns MD simulation.

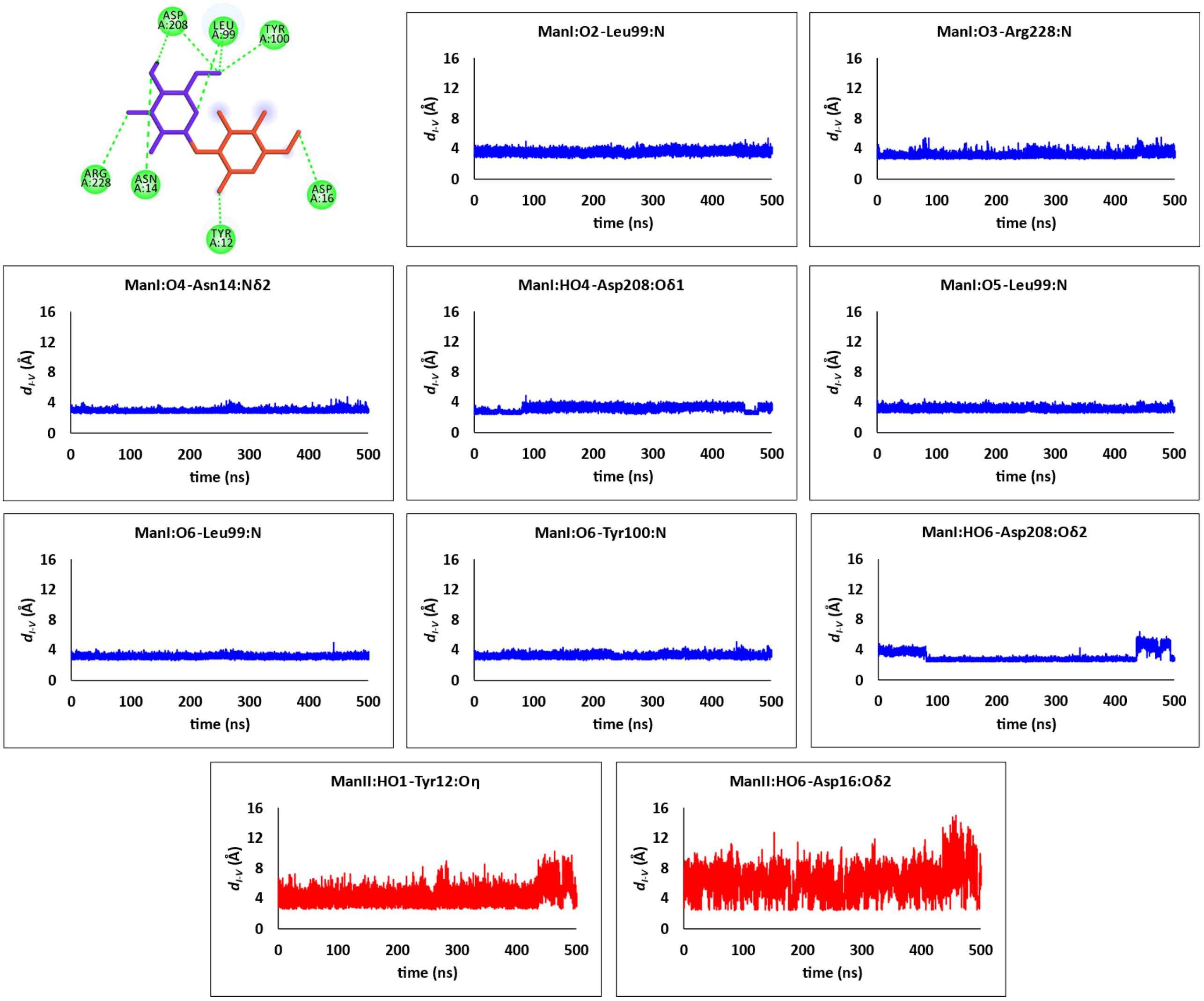

Figure S6 Time series for distance between selected atoms of α- n-α-(1→6)-[Man-α-(1→3)]-mannose (Ligand **4**) and conA over 500 ns MD simulation.

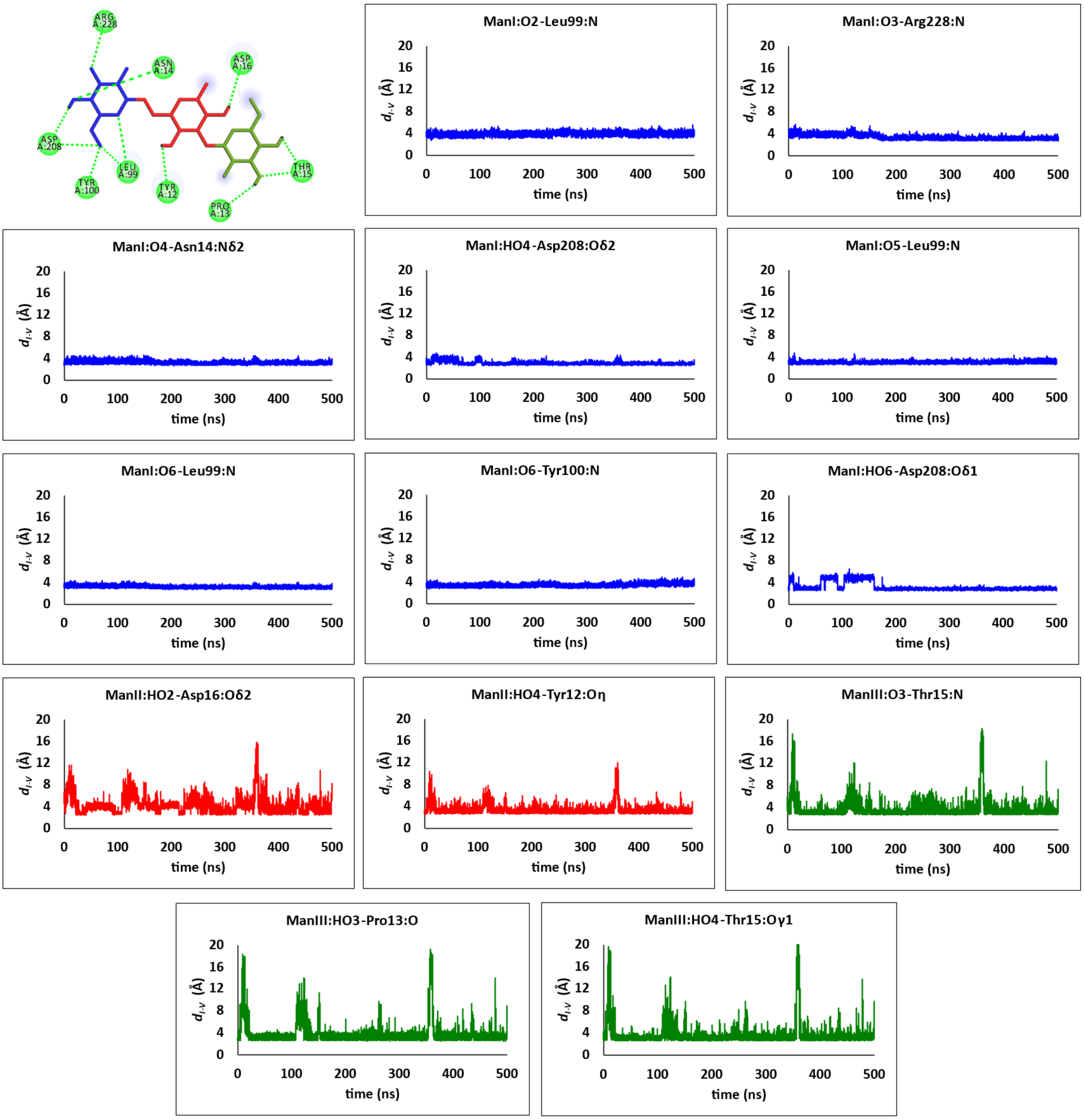

Figure S7 Time series for distance between selected atoms of β-GlcNAc-(1→2)-α-Man-(1→3)-[β-GlcNAc-(1→2)-α-Man-(1→6)]-Man (Ligand **5**) and conA over 500 ns MD simulation.

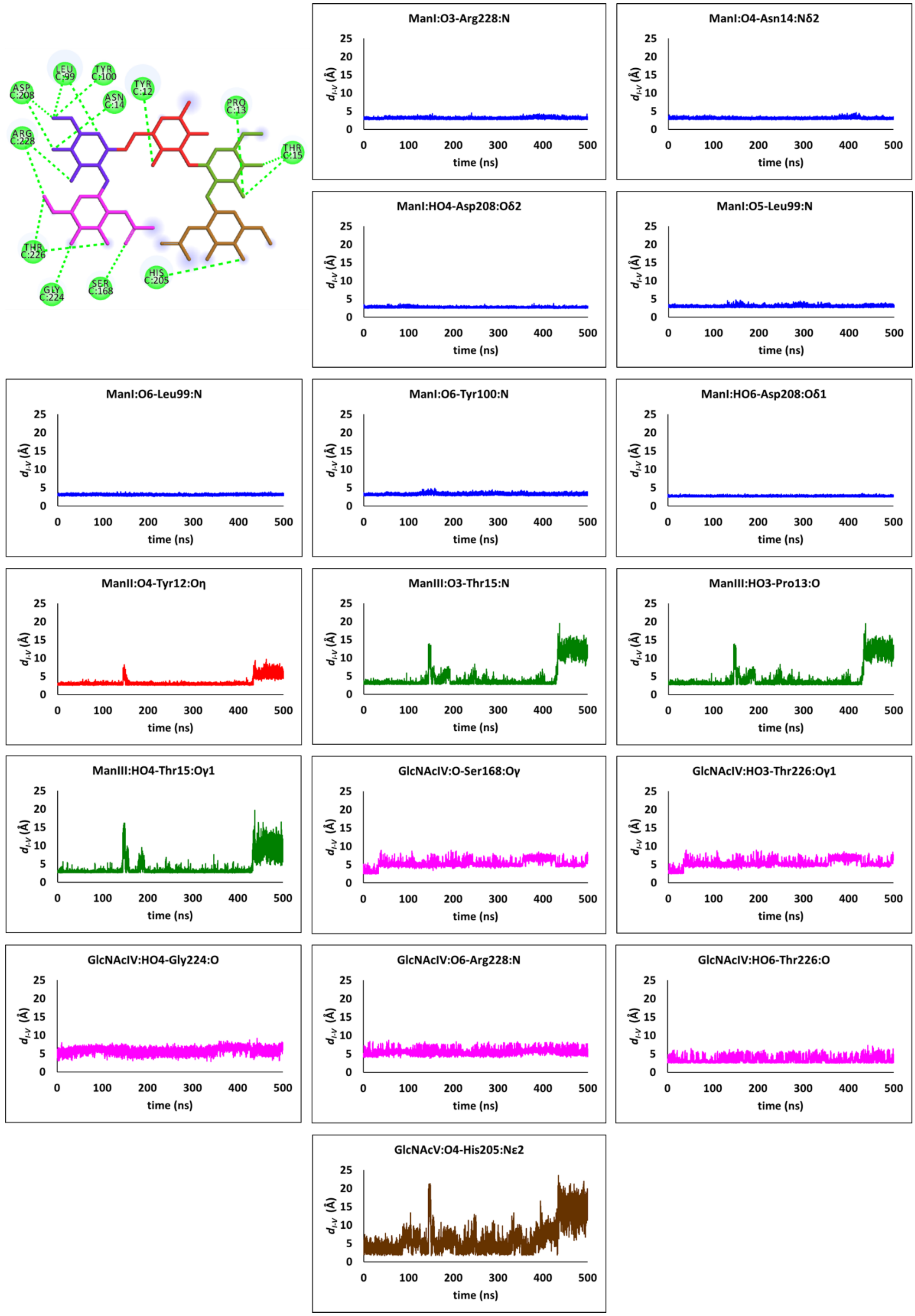

Figure S8 (a) Variation in binding free energies calculated for five replicas of ligands **1- 5** (in kcal/mol). Black and grey lines are for free energies from complex and solvent legs of free energy cycle respectively. (b) Variation in total ABFE calculated for five replicas of each complex. Error bars from MBAR estimate.

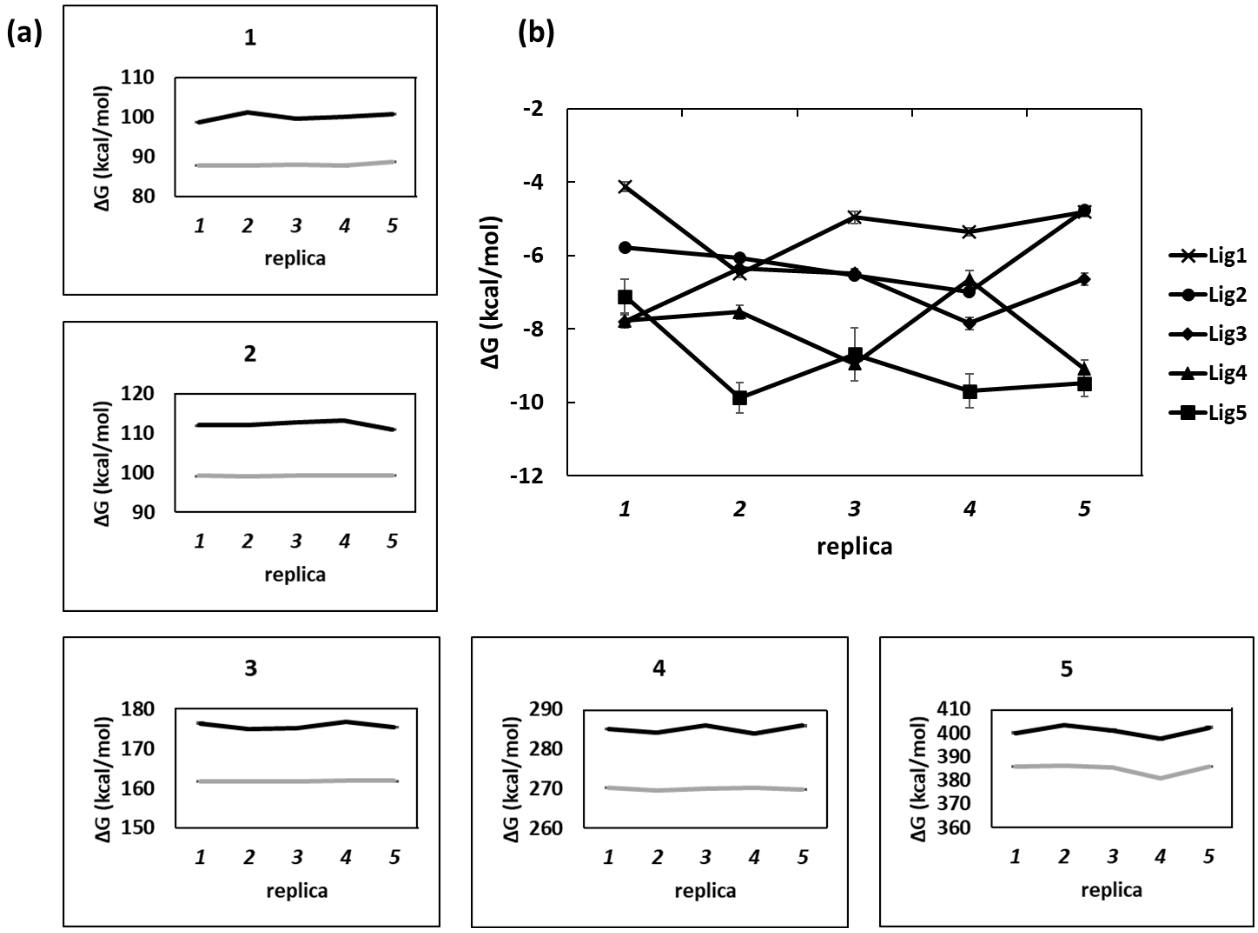

Figure S9 (a) Starting frames of the 5 replicas run for ligands **1 - 5** superposed to their crystal structure. (b) Starting frame of each replica of ligands **1 - 5** respectively, each superposed to its crystal pose. The carbon atoms of the crystal pose are colored green, while the carbon atoms of replicas *1*, *2*, *3*, *4*, and *5* are colored magenta, orange, gold, navy blue, and grey respectively. Numbering on figure refers to ligand not replica. Hydrogen atoms removed for clarity.

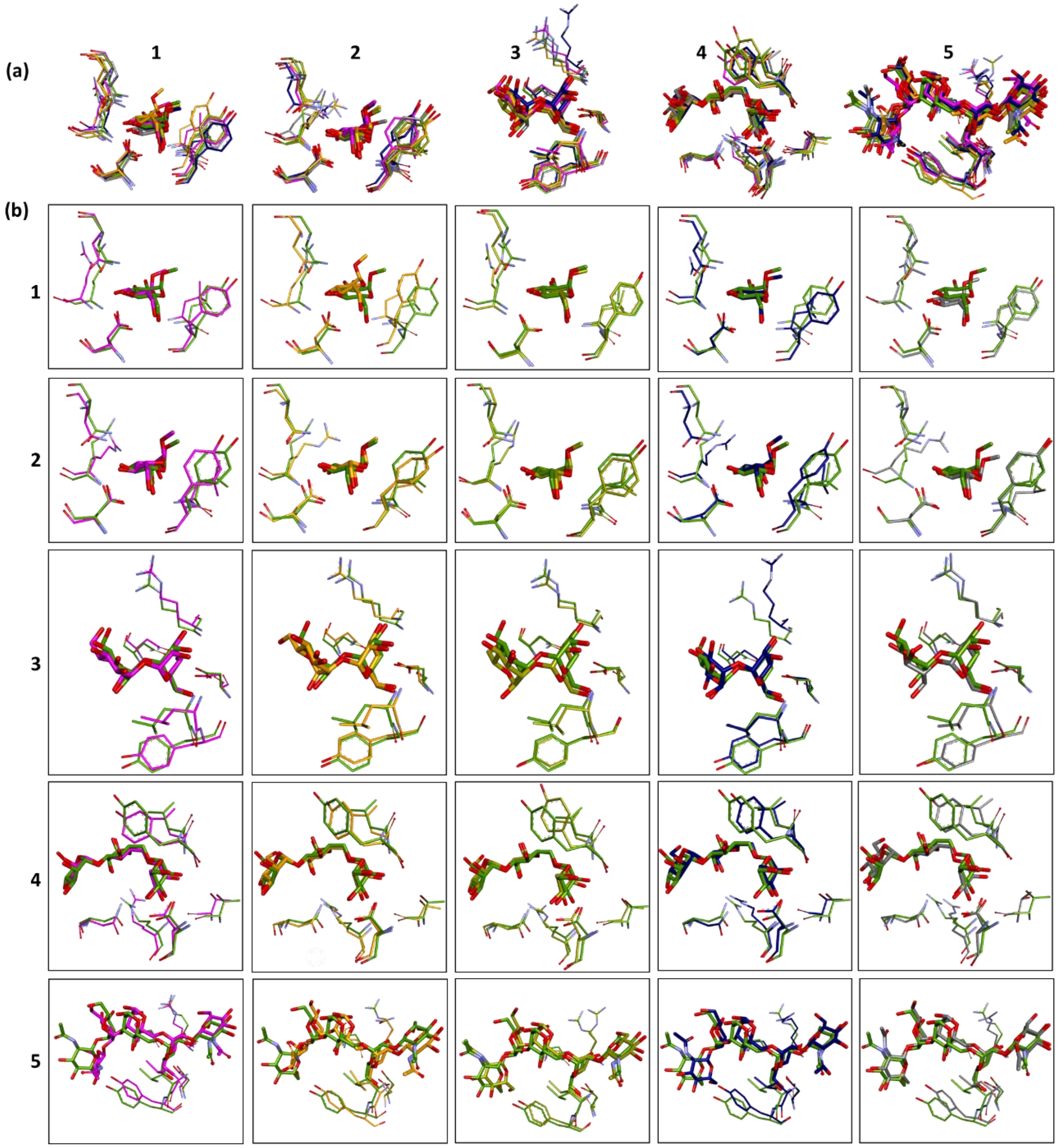

Table S1 Calculated binding free energies for complexes of ligands **1 - 5** over five replicas (in kcal/mol). Range in computed ΔG_{bind} sampled by five replicas also shown. Experimental binding free energies and binding efficiencies of ligands (free energy/number of heavy atoms) also shown. Uncertainties of calculated energies per replicas is uncertainty of MBAR free energy estimate, while that of $\Sigma\Delta G_{calc}$ is standard deviation.

	ΔG_{calc}				
**Ligand**	**1**	**2**	**3**	**4**	**5**
**Replica**					
**1**	-4.13 ± 0.13	-5.78 ± 0.11	-7.80 ± 0.18	-7.78 ± 0.18	-7.11 ± 0.46
**2**	-6.50 ± 0.10	-6.07 ± 0.08	-6.34 ± 0.17	-7.54 ± 0.19	-9.87 ± 0.42
**3**	-4.96 ± 0.16	-6.53 ± 0.11	-6.49 ± 0.14	-8.95 ± 0.16	-8.69 ± 0.73
**4**	-5.36 ± 0.11	-6.99 ± 0.10	-7.85 ± 0.17	-6.65 ± 0.25	-9.69 ± 0.47
**5**	-4.81 ± 0.12	-4.77 ± 0.09	-6.64 ± 0.16	-9.09 ± 0.25	-9.48 ± 0.35
**Min ΔG_{calc}**	-6.50 ± 0.10	-6.99 ± 0.10	-7.85 ± 0.17	-9.09 ± 0.25	-9.87 ± 0.42
**Max ΔG_{calc}**	-4.13 ± 0.13	-4.77 ± 0.09	-6.34 ± 0.17	-6.65 ± 0.25	-7.11 ± 0.46
**Range**	2.37	2.22	1.51	2.44	2.76
**$\Sigma\Delta G_{calc}$**	-5.15 ± 0.87	-6.03 ± 0.84	-7.02 ± 0.74	-8.00 ± 1.02	-8.97 ± 1.13
**ΔG_{exp}**	-4.49 ± 0.03	-5.33 ± 0.03	-6.30 ± 0.02	-7.54 ± 0.05	-8.38 ± 0.08
**Ligand efficiency$_{expt}$**	-0.35	-0.41	-0.27	-0.22	-0.14
**Ligand efficiency$_{calc}$**	-0.40	-0.46	-0.30	-0.24	-0.14

Table S2 Time-average number of hydrogen bonds for each of the hydrogen bond forming groups in ligands **1-5**

Ring	Atom	1		2		3		4		5	
		Protein	Solvent	Protein	Solvent	Protein	Solvent	Protein	Solvent	Protein	Solvent
I	O1	0.00	0.51	0.00	0.43	-	-	-	-	-	-
I	OH2	0.01	1.83	0.47	1.09	0.10	1.04	0.06	1.13	0.00	0.00
I	OH3	0.82	0.99	0.97	0.75	1.02	0.68	1.17	0.41	0.95	0.06
I	OH4	2.01	0.06	2.30	0.01	2.14	0.02	1.83	0.09	2.19	0.00
I	O5	0.62	0.00	0.53	0.00	0.85	0.00	0.92	0.00	0.89	0.00
I	OH6	2.12	0.01	1.90	0.00	1.82	0.00	1.79	0.00	1.99	0.00
		5.58	3.40	6.17	2.28	5.93	1.74	5.77	1.63	6.02	0.06
II	OH1	-	-	-	-	0.14	1.36	0.00	1.65	0.01	1.63
II	OH2	-	-	-	-	0.00	0.02	0.80	1.22	0.89	1.27
II	OH3	-	-	-	-	0.02	1.87	0.00	0.28	0.00	0.24
II	OH4	-	-	-	-	0.07	1.76	0.60	0.98	0.68	1.04
II	O5	-	-	-	-	0.15	0.20	0.01	0.71	0.03	0.63
II	OH6	-	-	-	-	0.54	1.56	0.00	0.21	0.00	0.20
						0.92	6.77	1.41	5.05	1.61	5.01

Table S2 Continued …

Ring	Atom	1		2		3		4		5	
		Protein	Solvent	Protein	Solvent	Protein	Solvent	Protein	Solvent	Protein	Solvent
III	OH2	-	-	-	-	-	-	0.02	1.74	0.00	0.20
III	OH3	-	-	-	-	-	-	1.17	0.81	1.28	0.70
III	OH4	-	-	-	-	-	-	0.82	0.83	0.86	0.80
III	O5	-	-	-	-	-	-	0.00	0.39	0.00	0.26
III	OH6	-	-	-	-	-	-	0.03	2.06	0.03	2.04
								2.04	5.83	2.17	4.00
IV	OH3	-	-	-	-	-	-	-	-	0.19	1.45
IV	OH4	-	-	-	-	-	-	-	-	0.00	1.76
IV	O5	-	-	-	-	-	-	-	-	0.00	0.00
IV	OH6	-	-	-	-	-	-	-	-	0.76	0.81
IV	NH	-	-	-	-	-	-	-	-	0.67	0.08
IV	O									0.05	1.11
										1.67	5.21

Table S2 Continued …

Ring	Atom	1		2		3		4		5	
		Protein	Solvent	Protein	Solvent	Protein	Solvent	Protein	Solvent	Protein	Solvent
V	OH3	-	-	-	-	-	-	-	-	0.00	1.95
V	OH4	-	-	-	-	-	-	-	-	0.03	1.62
V	O5	-	-	-	-	-	-	-	-	0.00	0.30
V	OH6	-	-	-	-	-	-	-	-	0.10	1.71
V	NH	-	-	-	-	-	-	-	-	0.00	0.79
V	O	-	-	-	-	-	-	-	-	0.18	1.09
										0.31	7.46
**Total**		**5.58**	**3.40**	**6.17**	**2.28**	**6.85**	**8.51**	**9.22**	**12.51**	**11.05**	**21.74**

Table S3 Calculated binding free energies for chains A and B of the complexes of ligands **2** and **5** over five replicas (in kcal/mol). Range in computed ΔG_{bind} sampled by five replicas also shown. Experimental binding free energies also shown. Uncertainties of calculated energies per replicas is uncertainty of MBAR free energy estimate, while that of $\Sigma\Delta G_{calc}$ is standard deviation.

ΔG_{calc}		
**Ligand**	**2A**	**5B**
**Replica**		
**1**	-6.16 ± 0.09	-10.59 ± 0.36
**2**	-7.92 ± 0.10	-12.99 ± 0.43
**3**	-6.78 ± 0.09	-9.99 ± 0.39
**4**	-6.76 ± 0.09	-9.40 ± 0.40
**5**	-7.71 ± 0.08	-10.55 ± 0.50
**Min ΔG_{calc}**	-7.92 ± 0.10	-12.99 ± 0.43
**Max ΔG_{calc}**	-6.16 ± 0.09	-9.40 ± 0.40
**Range**	1.76	3.59
**$\Sigma\Delta G_{calc}$**	-7.07 ± 0.73	-10.70 ± 1.37
**ΔG_{exp}**	-5.33 ± 0.03	-8.38 ± 0.08
**Signed error**	-1.74	-2.32
