# Supplementary material for: Analysis of Glycan Recognition by Concanavalin A Using Absolute Binding Free Energy Calculations
Source: J Chem Inf Model. 2024 Oct 16;64(20):8063–73. doi: 10.1021/acs.jcim.4c01088 (PMC11523069; doi:10.1021/acs.jcim.4c01088)
Supplement: Supplementary file 2 — ci4c01088_si_002.pdf [file ci4c01088_si_002.pdf]

SMILES strings for ligands **1** – **5**:

1. CO[C@H]1O[C@H](CO)[C@@H](O)[C@H](O)[C@H]1O
2. CO[C@H]1O[C@H](CO)[C@@H](O)[C@H](O)[C@@H]1O
3. OC[C@H]1O[C@H](O[C@H]2[C@@H](O)[C@H](O)[C@@H](CO)[O]=C2O)[C@@H](O)[C@@H](O)[C@@H]1O
4. OC[C@H]1O[C@H](OC[C@H]2O[C@H](O)[C@@H](O)[C@@H](O[C@H]3O[C@H](CO)[C@@H](O)[C@H](O)[C@@H]3O)[C@@H]2O)[C@@H](O)[C@@H](O)[C@@H]1O
5. CC(=O)N[C@@H]1[C@@H](O)[C@H](O)[C@@H](CO)O[C@H]1O[C@H]2[C@@H](O)[C@H](O)[C@@H](CO)O[C@@H]2OC[C@H]3O[C@H](O)[C@@H](O)[C@@H](O[C@H]4O[C@H](CO)[C@@H](O)[C@H](O)[C@@H]4O[C@@H]5O[C@H](CO)[C@@H](O)[C@H](O)[C@H]5NC(=O)C)[C@@H]3O
